# Medicine procurement in hospital pharmacies of Nepal: A qualitative study based on the Basel Statements

**DOI:** 10.1371/journal.pone.0191778

**Published:** 2018-02-05

**Authors:** Mina Shrestha, Rebekah Moles, Eurek Ranjit, Betty Chaar

**Affiliations:** 1 Faculty of Pharmacy, The University of Sydney, Sydney, New South Wales, Australia; 2 World Hospital Pharmacy Research Consortium (WHoPReC); 3 Department of Pharmacology, Kathmandu Medical College, Kathmandu, Bagmati, Nepal; Drake University College of Pharmacy and Heath Sciences, UNITED STATES

## Abstract

**Background:**

Accessibility and affordability of evidence-based medicines are issues of global concern. For low-income countries like Nepal, it is crucial to have easy and reliable access to affordable, good-quality, evidence-based medicines, especially in the aftermath of natural or manmade disasters. Availability of affordable and evidence-based high quality medicines depends on the medicine procurement procedure, which makes it an important aspect of healthcare delivery. In this study, we aimed to investigate medicine procurement practices in hospital pharmacies of Nepal within the framework of International Pharmaceutical Federation [FIP] hospital pharmacy guidelines “the Basel Statements”.

**Method:**

We conducted semi-structured interviews with hospital pharmacists or procurement officers in hospital pharmacies of four major regions in Nepal to explore procurement practices. Data were collected until saturation of themes, analysed using the framework approach, and organised around the statements within the procurement theme of the Basel Statements.

**Results:**

Interviews conducted with 53 participants revealed that the procurement guidelines of the Basel Statements were adopted to a certain extent in hospital pharmacies of Nepal. It was found that the majority of hospital pharmacies in Nepal reported using an expensive direct-procurement model for purchasing medicines. Most had no formulary and procured medicines solely based on doctors’ prescriptions, which were heavily influenced by pharmaceutical companies’ marketing strategies. Whilst most procured only registered medicines, a minority reported purchasing unregistered medicines through unauthorised supply-chains. And although the majority of hospital pharmacies had some contingency plans for managing medicine shortages, a few had none.

**Conclusions:**

Procurement guidelines of the Basel Statements were thus found to be partially adopted; however, there is room for improvement in current procurement practices in hospital pharmacies of Nepal. Adoption and regulation of national and international policies is recommended for enhancing medicine accessibility, as well as improving preparedness for health emergencies during natural disasters and health epidemics.

## Introduction

A hospital pharmacy is an integrated part of healthcare in health institutions and is responsible for all medicine-related and management health processes to optimize outcomes and enhance the safety and quality of health services provided to patients [1]. There is large variation in the nature of services provided at hospital pharmacies around the globe, ranging from basic supply to advanced clinical services. However, with growing focus on patient-centred care, more complex services have become necessary in hospital pharmacies in many countries. In order to enhance hospital pharmacy practice globally, standardised guidelines based on essential elements of hospital pharmacy practice need to be followed [2, 3].

With the objective of developing global consensus statements for advancement of hospital pharmacy practice, the Hospital Pharmacy Section of International Pharmaceutical Federation (FIP) developed the first set of international consensus statements, named the Basel Statements, in 2008 [4]. The Basel Statements are 75 statements grouped under six key elements of hospital pharmacy practice, and are considered valuable guidelines for standardizing hospital pharmacy practice around the globe. Medicine Procurement is one of the six themes covered by the Basel Statements [4].

The procurement theme of the Basel Statements (Table 1) highlights different aspects of procurement, such as a) implementation of an appropriate and cost-effective procurement model with reliable information facilities and necessary funds to facilitate transparent and ethical purchasing, b) medicine selection based on formulary by adopting strong quality assurance principles, and c) establishment of contingency plans for effective management of medicine shortages [4].

**Table 1:** Procurement guidelines of the Basel Statements 2008.

STATEMENT NUMBER	STATEMENTS
**17**	The procurement process must be transparent, professional, and ethical to promote equity and access and to ensure accountability to relevant governing and legal entities.
**18**	Procurement should be guided by the principle of procuring for safety.
**19**	Procurement of pharmaceuticals is a complex process that requires pharmacist control and technically competent staff.
**20**	Operational principles for good procurement practice should be regularly reviewed and procurement models adapted to fit different settings and emerging needs in the most appropriate and cost effective way.
**21**	Procurement must be supported by strong quality assurance principles to ensure that poor quality medicines are not procured or allowed into the system. Proper storage to ensure maintenance of quality in the whole supply pipeline is mandatory.
**22**	Procurement should not occur in isolation, but rather be informed by the formulary selection process.
**23**	Good procurement must be supported by a reliable information system that provides accurate, timely, and accessible information
**24**	A formal mechanism must be in place for pharmacists to request designated funds to procure medicines for their patients.
**25**	Each pharmacy should have contingency plans for medicines shortages and purchases in emergencies.

Pharmaceutical procurement is a multi-disciplinary process requiring medical, pharmaceutical, managerial, financial and often political expertise An effective pharmaceutical procurement process should ensure availability of the right drugs in the right quantities, at the right time, for the right patients at reasonable prices, and at recognizable standards of quality [5].Pharmaceutical procurement is very susceptible to unethical practices. A study undertaken in 2009 demonstrated that transparency, professionalism and equity are some of the major concerning issues related to procurement in healthcare settings [6]. Although a global problem, multiple predisposing factors such as having a weak regulatory authority, lack of regulation enforcement, low staff remuneration, poor procedures and inadequate payment practices, place developing countries at higher risk of corruption [6]. According to Corruption Measurement Tools of the Transparency International, Nepal is vulnerable to corruption with low corruption perceptions index of 31 out of 100 and corruption control score of -0.68 (score ranges from -2.5 to 2.5) [7]. Lack of transparency can have negative health and economic consequences which can lead to loss of credibility and clients’ trust in the hospital services [5]. Moreover, this can have greater impact on poorer people, because they can neither afford these consequences nor opt for any alternatives, depriving them of access to medicines [8].

Rational selection of medicines is also considered one of the important factors associated with medicines use [9]. Data from the World Health Organization (WHO) shows that more than half of all medicines prescribed, dispensed or sold are inappropriate [10]. Moreover, medicine selection processes have been influenced by marketing strategies of pharmaceutical industries that manipulate scientific evidence in favor of newer, more expensive, on-patent drugs. For example, pharmaceutical companies can even influence physicians practicing in developed countries like United States of America (USA) to add medicines onto the formulary [11]. Such influential practices have also been seen in Nepalese hospitals [12].

Clinical and pharmaceutical advancements and innovations around the world are causing incremental increases in medicine and total health expenditure [13]. These increases have greater impact in developing countries like Nepal, which have limited resources and lack of reimbursement for medicines, resulting in the necessity for the general public to pay entirely by themselves [14, 15].

Additionally, the quality of medicines is one of the key components of pharmaceutical procurement because of increasing availability of counterfeit, substandard and contaminated medicines on the market, that have the potential to pose serious health threats [15]. A survey by WHO showed that the majority (63%) of counterfeit medicines came from East Asian countries, with India (which is a neighboring country of Nepal) being the leader of counterfeit medicines production (35%) [16]. Such problems are more common in developing countries like Nepal because of their weak regulatory policies and enforcement capacities as compared to developed countries with strong enforcement of regulatory policies and more transparent supply chains [17]. Counterfeit medicines account for 15–50% of all available medicines in low- and middle-income countries and tend to be more affordable [18]. Counterfeit and substandard medicines can have serious health and economic consequences, creating a never-ending cycle of poverty in the developing world [19].

Medicine shortages is another challenging issue that can have serious implications on healthcare delivery, economy of the hospital and patient’s health [20]. There are many contributing factors that can cause medicines shortages, but the major causes that can arise from within healthcare institutions are poor inventory management, lack of information flow and communication, changes in clinical practice, increase in demands, and natural disasters [20]. Health institutions in Nepal often face shortages of essential medicines, especially in remote part of the country [21], and during natural disasters like the devastating earthquakes of April and May 2015 [22] and health epidemics such as the repeated emergence of Cholera over several years [23].

More than half the population of low-income countries in Africa and Asia do not have regular access to essential medicines, either due to absence of regulatory authorities or limited capacity to regulate medicine distribution [5]. Medicines are the primary vehicle for healthcare delivery and have huge impact on the health and well-being of people around the globe. Therefore, equitable access to medicines is considered one of the fundamental rights of people and vital for achieving several Millennium Development Goals such as minimizing child death, improving maternal health and fighting against diseases like HIV/AIDS and tuberculosis[5, 24, 25]. With increase in adoption of contemporary evidence-based treatment systems, access to medicines has become an important topic for research globally [5, 6]. Since medicines cover large proportions of total health expenditures, ranging from 40% - 60% in developing countries [5, 6], access to health is affected by affordability of medicines [26]. Therefore, accessibility and affordability of essential medicines has also become a growing concern, especially in low and middle-income countries [5, 25, 27]. To promote health globally, WHO has attempted to promote access to essential medicines, especially to people of developing countries through the Essential Medicines List (EML) Program. Although this program has been hugely successful in improving accessibility of essential medicines in many low and middle-income countries, access to medicines is still a huge global problem, especially for the underprivileged population [18]. In 2011, a survey conducted on availability of essential medicines found that average availability of medicines is less than 60% in parts of South-East Asia and Africa. Due to poor availability of medicines in the public sector and lack of universal healthcare in many low-income countries, medicines tend to be unaffordable to the majority of population in these countries [26]. Although a number of barriers and solutions have been researched for promoting access to medicines, ranging from individual to national and international level initiatives, a common element among them has been medicine procurement [18, 26, 28, 29]. Availability of affordable and evidence-based high quality medicines depends on the procurement procedure of medicines and is therefore considered an important aspect of healthcare delivery [5].

Issues of accessibility and affordability of medicines are of paramount importance in Nepal [27], in particular, in light of the earthquakes that struck the country in April and May 2015, inflicting much damage on its infrastructure, finance and healthcare systems. Nepal is a low-income country in South-East Asia, ranking 145^th^ in the Human Development Index [30], with a poor health status as reported by the WHO [31]. Procurement practice in Nepal varies widely, depending on whether the health institution is governmental, a non-profit organization or a private institution. Procurement at public hospitals is conducted in three different ways: central push/pull system, district level drug programs, and community drug programs [32]. All public procurements are conducted in accordance with the Public Procurement Act 2007 and the Public Procurement Guidelines 2009 [33, 34], while private hospitals purchase their medicines from importers, wholesalers and retailers [32]. Although some information regarding procurement and distribution of some freely distributed essential medicines procured by the government and donor organizations are available [14, 32, 35], procurement practices across all types of health institutions of Nepal are still unknown. Therefore, investigating procurement in hospital pharmacies of Nepal based on international guidelines, namely the Basel Statements, is both practical and relevant for effectively improving access to affordable high-quality medicines. Although Nepal has a Public Procurement Act and Guidelines [33, 34], National Medicine Policy [36], and Hospital Pharmacy Directives [37], these policies and guidelines do not address medicine procurement in much detail. Moreover, the country does not have any specific medicine procurement-related policies and guidelines. Therefore, the FIP Basel Statements were selected as a framework for this study, as they are international guidelines that standardise global hospital pharmacy practices, and medicine procurement is one of the themes included.

This study aimed to investigate medicine procurement practices in hospital pharmacies of Nepal based on the international hospital pharmacy guidelines (FIP Basel Statements 2008) relating to medicine procurement.

## Method

### Ethics

The study was approved by the Human Research Ethics Committee at the University of Sydney [Project No. 2014/619]. All documents, including Participant Consent Form and Interview Guide were approved by the ethics committee. Written consent was obtained for each interview.

### Sampling

Hospital pharmacists or procurement officers at hospital pharmacies in private and public hospitals throughout Nepal were included in the study. Samples were collected using a passive snowballing technique. In the snowballing technique, informants refer prospective participants and these participants again refer other participants making a chain of referral [38]. Passive approach to snowballing was used for this study in which informants/participants first contacted the prospective participants requesting their participation in the study, and then upon agreement, investigators initiated communication and continued further interview processes.

The Hospital Pharmacists’ Association of Nepal, a private company of pharmaceutical products, and prominent figures in the profession such as the Vice President for the Hospital Pharmacy Section of International Pharmaceutical Federation (FIP) in the South East Asian Region were the key contact points for searching and reaching out to prospective participants. The only inclusion criterion was involvement of the participant in the procurement process. Hospital pharmacies that were based inside the premises of a hospital, and were run either by the hospital itself or leased by the hospital to a private organization, were included in the study. Hospitals from the five major regions in Nepal as well as those in major cities were targeted for inclusion in the study.

### Data collection and analysis

Semi-structured interviews were conducted with key stakeholders using a standard interview guide (S1 Appendix) based on procurement guidelines of the Basel Statements (Table 1) and assessment tools “Measuring Transparency in the Public Pharmaceutical Sector” [5] and “Operational Principles for Good Pharmaceutical Procurement” [39] published by WHO.

Interviews were conducted in either Nepali or English, based on the participant’s preference. All participants provided written consent. All recorded interviews were transcribed verbatim by the ground researcher [MS]. Transcripts of interviews conducted in Nepali were translated to English by a Nepali translator and verified by another Nepali co-author. All transcripts were verified by the interviewees before analysis. Each interviewee was posted or emailed transcripts for approval, and followed up to confirm reliability of the transcript. Data collection continued until saturation of themes occurred [40].

Data were analysed thematically utilising the framework analysis approach [41] based on the procurement theme of the Basel Statements [4], with assistance of NVivo10 software [42]. Framework analysis is an analytical approach, developed by Ritchie and Spencer (1980s), which organises data and develops a matrix mapped to key themes, concepts and categories (referred to as the “framework”) [43, 44]. This method is suitable for data covering similar topics or key issues that can generate themes capable of providing full description on the phenomenon under investigation [43, 45]. Framework analysis can be utilized in an either deductive or inductive way [45, 46]. A combined approach can be used when the research study already has some known issues (such as in our study: established medicine procurement methods) to further explore, and also aims to elicit any unanticipated aspects from the interviews (such as: different procurement procedures) [43]. Iterative analysis was conducted, initially by the ground researcher, and repeatedly discussed with the research team in continuous consultation. Each team member took several interviews to analyse separately. After five subsequent sessions of iterative analysis and discussion, the research team reached consensus on themes emerging. This iterative process further validated the reliability of the data.

## Results

Interviews were conducted with 53 participants; 52 were from hospital pharmacies (40 private, 12 public) in four different regions of Nepal. One participant worked at the Logistic Management Division under the Department of Health Services of the Ministry of Health and Population, and was responsible for procuring free supplies for public hospitals and associated health institutions of Nepal. Out of 53 participants, 46 were male and 7 female. As the central region of Nepal has several major cities and a larger number of hospitals, the majority of participants (n = 33) were from this region. Twelve participants belonged to the eastern region, 7 to the western region and 1 belonged to the far-western region (Table 2).

**Table 2:** Sample characteristics.

Sample	Sample Characteristics/Quantity
Participants	Hospital Pharmacists or Procurement Officers
Inclusion Criteria for Participants	Involvement in the procurement process
Sampling Technique	Passive Snow Balling
Key Contact Points	1. The Hospital Pharmacists’ Association of Nepal, 2. A private company of pharmaceutical products, and Prominent figures in the profession such as the Vice President of the Hospital Section of International Pharmaceutical Federation (FIP) for the South East Asian Region
Area Targeted	5 regions of Nepal
Area Covered	4 major regions of Nepal 1. Central Region: 33 2. Eastern Region: 12 3. Western Region: 7 4. Far-Western Region: 1
Public Hospitals	12
Private Hospitals	37+ 3 (International non-governmental, non-profit, organisation-funded hospitals)
Logistic Management Division, Department of Health Services, Ministry of Health and Population, Nepal	1
Male	46
Female	7
**Total Sample**	**53**

Our findings showed the existence of two types of hospital pharmacies, irrespective of the type of hospital: 1. those run by the hospital and 2. those run by a private organization under a lease agreement. Statements pertaining to the need for pharmacist-controlled procurement, information systems, existence of funding mechanisms, and proper storage conditions were all satisfactorily implemented, however, some guidelines were implemented only partially.

Notably, procurement practices were found to be affected by the nature of ownership of the hospital pharmacy: hospital-regulated hospital pharmacies tended to be more adherent to the Basel guidelines, while privately owned pharmacies deviated.

Utilising the Basel Statements relating to procurement processes, we organised the data under three main themes: Procurement Model, Medicines Selection and Contingency Plans. Below are the details of some important findings; all major findings are listed in Table 3.

**Table 3:** Major findings.

	FINDINGS	FREQUENCY	BASEL STATEMENTS	IMPLEMENTATION STATUS	BARRIERS
**A.**	**Theme 1: Procurement Model**				
**1)**	**Direct Procurement Model**	Majority	Basel Statements 17&20	Partial	
a)	Purchase of medicines directly from wholesalers at a price allocated by the manufacturers or wholesalers.				
b)	Selection of suppliers based on past relationships, incentives, and/or recommendations of pharmaceutical companies.				
c)	Absence of suppliers’ evaluation system				
d)	Follows regular procurement procedure but lacks well-defined written procurement procedure.				
**2)**	**Competitive Procurement Model**	Minority			Prescription-based selection and frequent change in prescription
a)	Purchase of medicines through either an open bidding process with predefined terms and conditions or competitive negotiations for achieving the best price for the medicines required. Pooled procurement followed by the Logistic Management Division of the Department of Health Services, Ministry of Health and Population, Nepal and the National Tuberculosis Centre (only those supplied by the Global Drug Facilities) for purchase of limited number of medicines that are distributed free-of-cost to patients.				Requirement of the competitive process such as lengthy and complex administrative process, larger order size, time and cost required for the bidding process.
b)	Selection of suppliers through a separate vendor selection committee on the basis of availability of medicines, assessment of services, quality and price of medicines, whether they were authorised providers, their legal record/status, and past work experiences.				
c)	Existence of suppliers’ evaluation system				
d)	Existence of well-defined written procurement procedure				
**3)**	**Appeal System**				
a)	Existence of complaints reporting system-Verbal	Majority			
b)	Existence of complaints reporting system-Written	Minority			
c)	Absence of complaints reporting system	Minority			
**4)**	**Expertise Involved**				
a)	Pharmacists	Majority	Basel Statement 19	High	
b)	Technically competent staffs (Trained pharmacy staffs)	Majority			
c)	Pharmacy Owner (not necessarily pharmacists/trained	Minority			
**5)**	**Information System**				
a)	Existence of information facilities	Majority	Basel Statement 23	High	
b)	Online sharing of procurement related information by Logistics Management Division of Department of Health Services, Nepal	One			
c)	Absence of any information facilities	Minority			
d)	Telecommunication as a mode of interactions with other procurement officers	Several			
e)	Existence of internal networking facilities	Minority			
**6)**	**Fund Request Mechanism**				
a)	Existence of fund request mechanism	All	Basel Statement 24	Full	
**B.**	**Medicine Selection**				
a)	Formulary Selection	Minority	Basel Statements 17, 18 & 22	Partial	Lack of formulary system and Pharmacy & Therapeutics Committee, Authority to doctors for medicine selection, and influence of aggressive pharmaceutical marketing
b)	Selection based on doctor’s prescription	Majority			
c)	Selection based on national Essential Medicine List (EML)	Majority			
d)	Procurement based on principle of procuring for safety	Minority			
e)	Selection of medicines based on quality and/or price of medicines	Minority			
f)	Influence of Aggressive Pharmaceutical Marketing	Majority			
g)	No influence of pharmaceutical marketing	Minority			
h)	Existence of Pharmacy and Therapeutic Committee	Minority			
i)	Restriction on interactions between health professionals and marketing representatives of pharmaceutical companies				
j)	Absence of declaration of conflict of interest by health professionals	Almost all			
	**Quality Assurance Principle**				
a)	Checking registration status of medicine	Majority	Basel Statement 21 and 18		
b)	Trusting doctor’s prescription and recommendations, and reputations of manufacturers	Minority		Moderate	
c)	Quality control system comprising of seeking analytical certificates and testing samples	Minority			
d)	Quality assurance based on clinical evidences or results	Minority			
e)	Absence of any quality assurance system	Minority			
f)	Existence of appropriate storage conditions	Minority			
g)	Lack of thermostatistically controlled temperature	Almost all			
h)	Availability of unregistered/ substandard/counterfeit medicine	Minority			
**C.**	**Contingency Plans**				
a)	Existence of strategies to manage medicine shortages which included brand substitution, emergency purchasing (from outside normal supply system, mainly from India), and borrowing from other pharmacies, controlling inventories and gathering prior information from suppliers and manufacturers	Majority	Basel Statement 25	Moderate	
b)	Existence of funds for emergency purchases	Majority			
c)	Absence of shortage management system	Minority			

### Theme 1: Procurement model

Our study revealed that hospital pharmacies in Nepal tended to use either one or both of two types of procurement models: 1.a direct-procurement model or 2.a competitive-procurement model. In the direct-procurement model, adopted by the majority of hospital pharmacies, medicines were purchased directly from wholesalers based on established business relationships, incentives, and/or recommendations of pharmaceutical companies, at prices allocated by manufacturers/wholesalers.

“If we buy from the supplier allocated by medical representatives, they will provide us with schemes and facilities. I want to maintain relationships and earn profit. Since price is always the same, we negotiate on facilities. When we will buy in bulk quantities they may offer us some extra facilities. Therefore, while purchasing medicine, bargaining will be done on such things.” [PP40]

On the other hand, some hospital pharmacies operated under the competitive-procurement model, following a well-defined written procedure, through either an open-bidding process with predefined terms and conditions or competitive negotiations for best prices.

“We call for registration of suppliers with predefined criteria. If the applying suppliers meet all those criteria, we will shortlist them through initial screening. Then we will ask for quotation from them and will select who offers us the best price.” [PP53]

These differences in procurement models were directly related to pharmacy ownership: the competitive-procurement model was typically adopted by hospital pharmacies owned by the hospital, while the direct-procurement model was implemented by privately-owned hospital pharmacies.

The study highlighted that pharmacists or trained technical staff were responsible for procurement processes in the majority of hospital pharmacies. In a few hospital pharmacies, the pharmacy owner, (not necessarily a pharmacist or trained) supervised procurement processes.

The study also revealed that procurement officers in most of hospitals utilized the internet, books (e.g. CIMS India; NIDS), company brochures and databases to gather information about medicines.

*“We have all sources; primary*, *secondary and tertiary sources*.*”* [PP18]

Some hospital pharmacies reported not having access to such resources.

Almost all interviewees reported that funds for procurement in their pharmacies were generated by sales of medicines. Where medicine sales were low, participants reportedly used credit or returned unused stock.

“We can request from the account section which collects bills of purchased medicine and money collected from the sales. We can also purchase medicine on credit from our regular suppliers.” [PP11]

### Theme 2: Medicines selection

Our study confirmed that the majority of hospital pharmacies did not have a formulary. Of the few that did adopt a formulary list, it was either devised by a formal Pharmacy and Therapeutic (P&T) Committee, or by an informal group of employees. An informal group of employees would be non-officials, mainly doctors or health professionals working in hospital, requesting or suggesting medicine purchases in a hospital as needed. Some procurement officers also mentioned considering the national EML while purchasing medicines.

Participants almost unanimously reported that doctors’ prescribing, irrespective of the formulary and EML, drove procurement.

“We have to keep medicines according to doctors’ prescriptions.” [PP30]

Additionally, almost all of the participating procurement officers reported that aggressive marketing strategies of pharmaceutical companies affected medicine selection, effectively creating undeclared conflicts of interest.

"Yes, there are influences. If you look at the whole of Nepal, there are influences. Since we don’t have Pharmacy and Therapeutic Committee, medicines are selected based on doctors’ prescriptions. Therefore, pharmaceutical companies have become successful in influencing doctors." [PP25]

One participant stated that some pharmaceutical companies donated to the hospital, and therefore it was desirable for procurement to be conducted through these companies.

We are practising in this way because we want to provide opportunities to all pharmaceutical companies. Sometimes, some companies may have invested in the hospital, and then we can discuss with even directors and can use medicines of such companies… Everyone’s interest is met. The pharmaceutical companies are also here for business… They conduct Continuing Medical Education (CME), conferences and other programs. Therefore, we will keep medicines from those companies on rotation basis ". [PP2]

Amidst the prescription-driven and commercially-influenced procurement practices, a small minority of hospital pharmacies confirmed that procurement was conducted in accordance with the Public Procurement Act 2007 without influence of companies.

"It is very transparent; 100% as per the law. Due to this Commission for the Investigation of Abuse of Authority (CIAA), things are very tough now. Public Procurement Act is there and we have to follow each and every step as per guidelines." [PP8]

Majority of hospital pharmacies showed no regulations or policies for managing influences of pharmaceutical companies.

"This is almost impossible in the context of Nepal because doctors are given more priorities and doctors are controlling the hospital rather than being controlled by hospital…Therefore, it is impossible to regulate doctors. This is more of a national issue rather than a hospital issue. The government has published Medicines Promotional Act for managing this and they can implement this act; then only will there be some control over this promotional strategy…otherwise they are free to continue with their marketing strategy." [PP4]

However, a few reported to manage influence by restricting pharmaceutical companies’ access to decision-makers.

"Medical representatives are not allowed to visit doctors. We have that rule to restrict medical representatives’ visit to our hospital; but meeting outside the hospital might happen." [PP50].

An interesting observation was that such hospital pharmacies tended to be run by the hospital and were often owned by the government or International Non-Governmental Organizations (INGOs).

On the issue of safety, only a few officers mentioned prioritising safety of medicines. Many pharmacists noted that they confirmed the registration status with national regulatory authorities before procuring.

"If medicines are registered in Department of Drug Administration (DDA) then we trust that they are of good quality. We only buy registered medicines.” [PP2]

However, a few hospital pharmacies, especially in cities near the Indian border, admitted that they had to procure unregistered medicines from unauthorised sources if prescribed by doctors or if medicines required were in shortage.

"Some medicines are important to patients and are life-saving medicines. Doctors have to prescribe such medicines and most of them are not available in Nepal. In such conditions when doctors prescribe them, we do purchase from India, even from black market. Such medicines are not kept here in the pharmacy; we stored them separately and even dispense secretly to patients. It is illegal but we are saving patients’ lives." [PP44]

When asked about adoption of a quality assurance system for controlling entry of substandard medicines, some respondents answered that they trusted doctors’ prescriptions and recommendations, and/or reputations of the manufacturers for ensuring quality of medicines.

"No, we don’t do that. If doctors prescribe then it must be of good quality. If they prescribe then we will sell anything, even stones." [PP20]

A few participants had a system in place for quality control, including seeking certificates of analysis and/or testing of samples.

"There is a pre-shipment inspection, post-shipment inspection, checking of Good Manufacturing Practice and analysis certificates. We appoint a third-party inspection agency for inspection…It is done for each product and each company that won the bid for supply. There is post-shipment inspection at the time of delivery. We randomly select medicines and send to national medicines laboratories for inspection" [PP15]

The study revealed that medicines in most hospital pharmacies were stored in appropriate conditions. Many had facilities for maintaining room temperature (20–25°C) and other conditions necessary to preserve the quality of medicines during storage.

### Theme 3: Contingency plans

Our results showed that the majority of pharmacists used brand-substitution, emergency-purchasing (from outside the normal supply system, mainly from India), and borrowing from other pharmacies, as strategies to manage medicine shortages.

"We will borrow from other hospital pharmacies or request suppliers to manage medicines for us. If it is not possible to manage from these sources then we will go for second option. We will discuss with doctors and substitute. We also control inventories and try to manage stock.” [PP52]

Several hospital pharmacists also claimed that they had a separate emergency fund, for pricier substitutions needed during emergencies. A few pharmacists stated that they did not have a shortage management system in place.

“If medicines are not available then we have to say no and request patients to search in other places.” [PP13]

Some hospital pharmacies managed inventories by stockpiling up to 3 months’ worth of medicines based on past consumption or seasonal changes and epidemics, as well as gathering prior information from suppliers/manufacturers to help manage medicine shortages.

“We usually maintain stock for 3 months so shortage does not happen that frequently. Sometimes supplier will inform us about upcoming shortages and ask whether we want to order in larger quantities. And in such conditions, we will order in larger quantity.” [PP14]

## Discussion

To our knowledge, this is the first study to explore procurement practices in hospital pharmacies in Nepal within the framework of the procurement guidelines of the Basel Statements. Our findings revealed mixed results regarding implementation of guidelines, which included partial adoption of guidelines in the majority of hospital pharmacies. This shows that, by and large, Nepalese hospital pharmacies have many good processes in place however there is room for improvement. Accordingly, several recommendations are proposed.

It is important to note that compliance with the Basel Statements may not always be in the best financial interests of private pharmacy ownership. This could therefore have contributed or explained lower compliance with the guidelines by some privately owned hospital pharmacies. Given the apparent correlation between pharmacy ownership and guidelines implementation, enforcement of the Nepalese National Health Policy 2014 [47] and Hospital Pharmacy Directives 2013 [37] could be an effective way to improve procurement practice and guidelines implementation. These require hospital pharmacies to be run by hospital administrations.

### Theme 1: Procurement model

The study found that the majority of hospital pharmacies followed the direct-procurement model, which is considered one of the most expensive models by WHO [48], and is thus inconsistent with Basel Statement 20 [4] and the Hospital Pharmacy Directives 2013 of Nepal [37]. Moreover, lack of well-defined procurement procedures and transparency in supplier selection is also in contrast to Basel Statements 17 and 20, which demand transparent, professional and ethical procurement practices [4]. According to the WHO, the restricted tender procedure, open for only prequalified suppliers, is considered the best procurement model for small countries like Nepal [48]. A small minority of hospital pharmacies adopted a competitive-procurement model, similar to other low-and middle- income countries like India and those in the Western Pacific region [49]. However the open tender system without pre-/post-bidding evaluation of suppliers is not in accordance with WHO recommendations [48].

Furthermore, barriers to utilization of the tendering process were identified, such as size of purchase order, stock levels, and administrative workloads, which are similar issues reported by WHO [39] and Arney [50]. To overcome these barriers, pooled procurement which creates buyers’ cartel for achieving price reductions, improved quality assurance and minimization of corruption has been recommended [49]. In particular, pooled-procurement with contractual agreements could be adopted [50, 51]; such models are successfully implemented in countries like the USA, Mexico, Chile and within United Nations Organizations [50]. This achieves competitive uniform pricing through a centralized tender system, allowing decentralized purchasing of smaller repeated orders and delivery to associated healthcare systems [50]. Additionally, several other cost containment strategies such as conducting pharmaco-economic evaluation, establishing prescribing guidelines and promoting use of generics could be utilized by procurement officers while purchasing medicines for hospitals [49, 52].

Although the Nepalese National Good Pharmacy Practice Guidelines 2005 [53] insists on pharmacist-controlled procurement, in line with Basel Statement 19, this was not evident in some hospital pharmacies. The main reason for the absence of pharmacist-controlled procurement was the nature of ownership of hospital pharmacy. As some hospital pharmacies in Nepal are owned by an individual or a group of persons, owner/s themselves wanted to procure medicines in order to maximize their profits and business.

Furthermore, and in contrast to the Basel Statements, the Nepalese Hospital Pharmacy Directives 2013 [37] do not specify this requirement. Better alignment of directives with guidelines should be ensured in the future. This necessitates education on the role of pharmacists in the procurement process and inclusion of pharmacist-control in Hospital Pharmacy Directives. Moreover, a multidisciplinary approach involving expertise from clinical pharmacology, pharmacoepidomology and pharmacoeconomics has also been recommended for improving procurement practices [13].

Reliance on unreliable sources of evidence-based healthcare was also a concern. Utilization of readily available online information sources provided by national and international organisations providing evidence-based guidelines [e.g. DDA, and WHO] should be encouraged. Finally, procurement officers should be educated about international guidelines developed by WHO and other international pharmaceutical associations [such as FIP] to improve their procurement practices.

### Theme 2: Medicines selection

Another challenging issue in pharmaceutical procurement was medicine selection. This study found that the majority of hospital pharmacies in Nepal are being operated without a hospital formulary to facilitate patient care and medicine procurement. This finding suggests that the Basel Statements 18, 21 and 22, the WHO [9], American Society of Health System Pharmacists (ASHP) [54] and Nepalese Hospital Pharmacy Directives (2013) [37] regarding adoption of a formulary system, were not followed. Hospital pharmacies in Nepal procured medicines based on prescription, and the majority of doctors did not utilise a formulary system to guide their prescribing behaviour. This is in contrast with medicine selection practices in the USA, Europe and Western Pacific countries that follow a formulary system [55]. Although a few hospital pharmacies reported the existence of a formulary or equivalent list, the selection process was not found to follow guidelines, possibly due to the absence of a formal pharmacy and therapeutics selection committee. However, while specific formularies were not found to be commonplace in this study, the majority of procurement officers did aim to maintain stock of medicines included on the national EML. This indicated some compliance with the procedures recommended by the Nepal Pharmacy Council [53] and WHO [48].

Further, occurrences of undeclared relationships between decision-makers and suppliers/manufacturers were also uncovered in this study. The lack of a Pharmacy and Therapeutic (P&T) Committee, authority of doctors in medicine selection, and influences of aggressive pharmaceutical marketing strategies were reported by interviewees as major barriers for formulary-based selection.

The dominance of doctors driving procurement based on what they prescribe without specific prescribing guidelines could be minimized by establishing a P&T Committee to develop formularies and add additional players in decision making processes [9] with the help of model formularies [national[56]; international [57]] and guidelines published by the WHO [9] and ASHP [54]. Introduction of hospital formularies can also be actively taken up by hospital pharmacists of Nepal.

The influence of pharmaceutical marketing is a heavily scrutinized topic around the world because of its impact on patient care. Influence of pharmaceutical companies on medicine selection and other procurement-related behaviours evident in Nepal is in contrast with Basel Statement 17 [4], which demands ethical procurement practice, and the Basel Statement 18 [4] which emphasizes on the safety of medicines. Such influential behaviours to induce prescription and sales of medicines have also been reported to pose enormous challenges in India [58], China [59] as well as developed countries like the US [11]. To address such issues, medical associations in the USA [60] and Australia [61] have attempted to manage relationships between medical professionals and pharmaceutical companies by implementing strict codes of ethics/conduct. Moreover, the American College of Clinical Pharmacy in the USA has developed guidelines for pharmacists to assist in managing ethical interactions with pharmaceutical industry [62]. On the contrary, the Code of Medical Ethics developed by the Nepal Medical Council [63] is yet to include any notes on ethical relationships between healthcare professionals and pharmaceutical companies. Although the DDA has developed guidelines on ethical promotion of medicines [64], our findings suggest that DDA has not been able to effectively enforce them. It is noteworthy that pharmaceutical companies in Australia [65] and the USA [66] also have their own codes of conduct that, among other guidelines, requires declaration of details of promotional activities to be disclosed. Nepalese pharmaceutical companies and regulatory authorities could develop similar codes to further promote ethical medicine use.

Furthermore, undeclared conflicts of interest observed in Nepalese hospital pharmacies suggest that there is room to decrease influence and bias. The WHO actually suggests establishing guidelines for declaration of conflict of interest by people involved in the decision-making processes in order to minimize corruption, favoritism or influence, and to increase transparency in procurement processes [5]. In line with this, Nepal could make regulatory and practical changes based on the WHO Good Governance for Medicines Medical Framework [67], which has achieved success in countries like Jordan, Thailand and Malaysia [67]. The Nepalese government could also introduce a reward-based system like that in the Philippines [68] and suggested by Ombaka [6] to encourage organizations to follow regulatory guidelines.

Interestingly, many participants did not seem to consider safety issues whilst procuring. Although most procurement officers aimed to procure registered medicines to ensure quality, a few reported procurement of unregistered medicines through unauthorised supply chains, especially from India, one of the leading countries for counterfeit and substandard medicine production [16]. Procurement of medicines from unauthorized sources raises concerns about the safety and efficacy of medicines. While it is understood that such procurement practices may only have been carried out under exceptionally dire circumstances, such emergency purchases should be made through the correct channels. The Department of Drug Administration in Nepal has formulated a special guideline [69] that allows special permission for procurement of unregistered medicines that are essential for patients. Hospital pharmacies could utilize this channel for purchasing emergency medicine of proven safety. In addition, regulatory authorities should also devise a mechanism to tackle unauthorised purchase of medicine.

Although a minority took the extra step of checking analysis certificates and performing analytical testing, trusting doctors’ prescriptions and the reputation of pharmaceutical companies in an environment of incentive-based selection and aggressive pharmaceutical marketing may not assure quality of medicine.

Procurement officers could utilize the model and guide of quality assurance system developed by the WHO [70] or the United States Pharmacopeia [71] to develop their own internal quality-assurance systems. Inexpensive and easy-to-use analytical test kits are also available to facilitate testing to minimize entry of substandard/counterfeit medicines [72].

In an attempt to combat counterfeiting globally, the WHO has developed a toolkit named “BE AWARE” [73] in collaboration with World Health Professionals Alliance. The WHO has also established guidelines for the development of measures to combat counterfeit drugs [74], which could be a useful resource. Nepal can also minimize the risk of purchasing poor quality medicines by utilizing the list generated by the WHO prequalification of medicines program [75], especially when purchasing from suppliers from countries with high risk of counterfeit medicine production.

### Theme 3: Contingency plans

The majority of hospital pharmacies reportedly managed medicine shortages through spontaneous workarounds, such as searching for alternatives, emergency purchasing, and borrowing from other hospital pharmacies. Some even stockpiled or tried to acquire prior knowledge about anticipated shortages, to help overcome shortages in epidemics or seasonal demands. These measures were in line with some guidelines recommended by ASHP [20], but possibly not sustainable or sufficient for managing and achieving preparedness for medicine shortages for the long term or times of emergency. The reported lack of shortage management systems in few hospital pharmacies indicated that Basel Statements 25 pertaining to the existence of contingency plans for medicine shortages was not fully addressed. Other countries such as Canada, USA and Europe, have regulatory frameworks, preparedness and strategic approaches in place to address medicine shortages [76]. Hospital pharmacies in Nepal would benefit from utilizing the three-phased approach consisting of identification and assessment, preparation, and contingency phases, proposed by ASHP for managing shortages of critical or essential medicines [20]. This allows for rationing and distributing available medicines ethically and purchasing medicines during emergencies with safety, quality and cost of medicine under consideration [20, 77]. Moreover, an information system similar to those of developed countries [76] could be utilized, or even made compulsory, as in the USA [78], for sharing information on shortages and management strategies.

Nepal recently endured disastrous earthquakes in April and May 2015 causing major medicines shortages, which was reported to be a crucial issue of concern in the massive aftermath of the tragedy [22]. In such circumstances, adoption of preparedness and strategic contingency plans would be of great significance for providing timely delivery of health services during emergencies. Hospital pharmacies, with active involvement of Ministry of Health and Population, should form a plan to address medicine shortages, as well as emergency management of medicines.

## Conclusions

The findings of this study indicated that procurement guidelines of the Basel Statements are currently implemented to a certain extent in hospital pharmacies of Nepal. This study also found some significant factors that influenced procurement practices in Nepal such as hospital pharmacy ownership, pharmaceutical companies’ influences, authority of doctors, and lack of regulatory enforcement. Moreover, purchase of unregistered medicines from unauthorised supply chain reported in the study showed possibility of health threats by counterfeit and substandard medicines. Absence of strategic contingency plans during shortage was also a major concern especially as Nepal could be heavily reliant on other countries for essential and lifesaving medicines in emergency situations such earthquakes, flooding, other natural disasters or health epidemics. Adoption and regulation of national and international policies is recommended for improving accessibility of high-quality, evidence-based and affordable medicines as well as preparedness for health emergencies during natural disasters and health epidemics. Enforcement of such regulations might be challenging both in terms of patient access and to actual enforcing–however, by highlighting findings of this study, we believe we can contribute to the general debate on access to medicines and challenges faced in Nepal.

## Strengths and limitations

This study successfully examined the medicine procurement practices of Nepal and has proposed evidence-based recommendations to improve current medicine procurement practices in Nepal. Although this qualitative study did not cover all hospitals of Nepal and thus may not be completely generalizable, major hospitals from most regions of Nepal were included and therefore provided meaningful insight into medicine procurement practices in Nepal. This study involved interviews with hospital pharmacists and procurement officers, however perspectives of policy makers, national regulatory authorities and hospital administrators could have provided in-depth and first-hand perspectives about the practical challenges in the adoption, regulation and implementation of guidelines. Further studies are recommended to include the perspectives of such key stakeholders.

Social desirability bias can also be common in this kind of research. To address potential bias, the ground researcher reassured all participants of anonymity and confidentiality. Nonetheless, we cannot guarantee that the data was completely void of social bias.

## Supporting information

S1 Appendix Interview guide.
(DOC)
