# Supplementary material for: Medicine procurement in hospital pharmacies of Nepal: A qualitative study based on the Basel Statements
Source: PLoS One. 2018 Feb 5;13(2):e0191778. doi: 10.1371/journal.pone.0191778 (PMC5798759; doi:10.1371/journal.pone.0191778)
Supplement: S1 Appendix — Interview guide. (DOC) [file pone.0191778.s001.doc]

# S1 Appendix: Interview Guide

1. How do you procure medicines in your hospital?

Prompts:

- 1. Does your hospital have Standard Operating Procedure for the procurement?
  2. Are the decisions made by selection committee publicly disseminated? If yes, where?
  3. Is the procurement of medicines based on evidence of safety? National essential medicines list or hospital formularies?
  4. Are pharmacists involved in the procurement procedure? If not, who is the responsible person for handling the procurement process?

1. Are there competitive procedures for the procurement of medicines?

If yes, prompts:

- 1. Are the contract specifications publicly available and distributed with tender documents?
  2. Are tenders and tender results publicized?
  3. Are there specific criteria for tender committee membership? Is the membership permanent?

1. Does your hospital have Pharmacy &Therapeutics Committee [PTC], formulary list, standard treatment guidelines and formulary manual?

If yes, prompt:

- 1. If a PTC is present, who are the members of the committee?
  2. Are there clear criteria for selection committee membership? If yes, what are they?
  3. Do terms of reference exist which describe the purpose of the selection committee, its composition, processes and duration?
  4. Are there clear rules for decision-making for the committee decisions? Is the decision made in a democratic manner?
  5. Is there a conflict of interest form that members of the selection committee and tender committee are obliged to complete? Are there clear sanctions for breach of these regulations?
  6. Is there a law or regulation prohibiting members of the drug selection committee from accepting support in kind or in cash from pharmaceutical companies?

1. Are there clear written criteria for adding and removing medicines for formulary list? If yes, what are they?
   1. Is the inclusion of medicines in the formulary list based on evidence of cost-effectiveness and health needs?
   2. Is there a clear algorithm, based on utilization of services and health needs to determine quantity and type of medicines purchased?
2. Is quality of medicines tested as a part of procurement procedure? If yes, which method do you follow? Prequalification or Post-qualification or both? Does your have own in-house quality control laboratories? Where are they tested?
3. Is supplier performance monitored annually?
4. Do your hospital storage facilities satisfy the desired storage conditions of all medicines? Are storage facilities accessible to all? If no, who is/are the authorized person/s for access?
5. What type of security system is present in your storage facilities? Manual or Electronic?
6. Does your hospital have information system for tracking the procurement procedure, communication exchange, international reference pricing and other useful resources?
7. Is there a management information system used to report product problems in procurement?
   1. Is the information obtained from monitoring used to influence future procurement decisions?
   2. How are medicines shortages managed in the hospital and how are they purchased during emergencies?
8. How is procurement of medicines funded in the hospital? Is there existence of any funding mechanism for requesting funds?
